# 

**Published:** 1918-07-20

**Authors:** 


To obtain **The HOSPITAL**
regularly **EVERY THURSDAY** a definite order **must** be placed with your Newsagent **at once.** All Newsagents, Booksellers, and Railway Bookstalls will gladly book your order, but copies cannot be stocked for sale to chance customers.

To obtain **The HOSPITAL** regularly **EVERY THURSDAY** a definite order **must** be placed with your Newsagent **at once.** All Newsagents, Booksellers, and Railway Bookstalls will gladly book your order, but copies cannot be stocked for sale to chance customers.

☛ A Trumpet Call to Nurses: The Gospel of Self-Help, by "Ierne," see p. 348; Matrons' and Sisters' Appointments, see p. 356.

### Matrons' and Sisters' Appointments.

Clapham Maternity Hospital.—Miss Lucie Howells has been appointed matron. She was trained at the Dudley Road Infirmary, Birmingham, and has been assistant matron and home sister at the Birmingham and Midland Hospital for Women.

Consumption Sanatorium of Scotland, Bridge of Weir.—Miss Eleanor Harvey has been appointed night sister. She was trained at Leeds Township Infirmary, later being staff nurse there, and at Leeds Sanatorium, Gateforth.

North Lonsdale Hospital, Barrow-in-Furness.—Miss Alys M. Hatton has been appointed theatre sister. She was trained at the Royal Salop Infirmary, Shrewsbury; has been night and ward sister at the General Hospital, Walsall; ward sister at the Miller Hospital, Greenwich; and sister at the Red Cross Hospital, Sandiway, Northwich.

**NOTE.—Particulars of Appointments for insertion in this column will be welcomed.**

